# Synergistic blend of natural essential oils improved growth performance and gut barrier integrity in broilers by alleviating intestinal inflammation

**DOI:** 10.1016/j.psj.2025.105783

**Published:** 2025-09-03

**Authors:** Vetriselvi Sampath, Lane Pineda, Ellen Hambrecht, In Ho Kim

**Affiliations:** a Department of Animal Biotechnology, Dankook University, Cheonan 31116, South Korea; b Smart Animal Bio Institute, Dankook University, Cheonan 31116, South Korea; c Trouw Nutrition, The Netherlands

**Keywords:** Intestinal health, Calprotectin, Gut health, Mucin, and Zona occludens genes, plant extracts

## Abstract

This study evaluated the effects of a synergistic blend of natural plant extracts based on oregano, clove, and cinnamon (Fytera Perform) on growth performance, intestinal health, immune status, carcass traits, and meat quality in broiler chickens. A total of 840 Ross 308 broilers (44.47 ± 0.41 g) were randomly assigned to two dietary treatments: a control group (CON) fed a basal corn-soy, antibiotic-free diet, and a treatment group supplemented with Fytera Perform at 25 g/ton (FP). Enteric inflammation was induced in all birds from days 22 to 28 by administering 2.5 % Dextran sodium sulfate via drinking water. Performance parameters were recorded throughout the study, while intestinal health parameters were measured on day 28 using two birds per replicate. Meat quality was assessed on day 28, and carcass traits were evaluated on day 42. Over the entire production period, broilers receiving FP supplementation demonstrated a 4.6 % increase in average daily gain and a 4.4 % reduction in feed conversion ratio (*P* < 0.05). On day 42, FP-supplemented birds exhibited a significantly greater body weight, weighing 112 g more than those in the CON group (*P* = 0.003*)*. Intestinal health was enhanced in the FP group, with lower fecal calprotectin levels, increased ileal villous height, and an elevated villous-to-crypt ratio (*P* < 0.05). Additionally, FP supplementation upregulated gene expression of mucin 2 and tight junction protein zona occludens-1, while downregulating pro-inflammatory cytokines IL-1β and IL-8 (*P* < 0.05). Meat quality parameters were also improved in the FP-supplemented group, with significantly lower drip loss at 5 days and 7 days of storage (*P* < 0.05). Moreover, the dressing percentage tended to be higher in the FP group compared to CON on day 42 (*P* = 0.07). These findings demonstrate that supplementation with a synergistic blend of plant extracts derived from oregano, clove, and cinnamon improves intestinal morphology, enhances gut barrier function, and reduces inflammatory responses, thereby promoting nutrient absorption, growth performance, meat quality, and overall health in broiler chickens.

## Introduction

Intestinal inflammation poses a significant challenge to modern broiler production, frequently occurring subclinically and negatively impacting growth performance, feed efficiency, and overall health (Fairman, 2024; Tellez et al., 2023). This condition is often triggered by a range of environmental and management-related stressors, including high stocking density, heat stress, dietary imbalances, and microbial dysbiosis, which activate pro-inflammatory signaling pathways, disrupt tight junction proteins, and impair mucosal integrity. These physiological disruptions compromise nutrient absorption and increase intestinal permeability, ultimately undermining productivity (Mishra and Jha, 2019; Kikusato and Toyomizu, 2023; Rostagno, 2020).

Maintaining intestinal barrier function is critical for optimal nutrient assimilation and productivity in poultry. The intestinal epithelium forms a selective barrier that facilitates nutrient uptake while preventing the translocation of pathogens and antigens. Disruption of this barrier, particularly in young birds, heightens susceptibility to infection and systemic inflammation, thereby further compromising growth (Abo-Al-Ela et al., 2021; Awad et al., 2017).

Nutritional strategies that enhance epithelial integrity and modulate immune responses represent promising approaches to mitigating intestinal inflammation under stress conditions (Kikusato and Toyomizu, 2023; Kogut, 2019). Phytogenic feed additives derived from medicinal plants, particularly essential oils from oregano, cinnamon, and clove possess antimicrobial, antioxidant, and anti-inflammatory properties (Ammar et al., 2021). These compounds have been shown to improve nutrient digestibility, modulate gut microbiota, and protect intestinal tissues from oxidative and inflammatory damage (Abd El-Aziz et al., 2024; Ciftci et al., 2010; Ding et al., 2022; Liyanage et al., 2021). Specifically, oregano oil supports mucosal integrity and microbial balance, whereas cinnamon and clove oils enhance digestive enzyme activity and improve intestinal morphology (Mohammadi et al., 2014; Najafi and Torki, 2010).

Despite growing interest and promising results, the efficacy of phytogenic feed additives under conditions of intestinal challenge remains underexplored, with only a few studies conducted to date (Blue et al., 2024; Hascoet et al., 2025). To effectively assess these additives under enteric stress, robust experimental models that replicate commercial conditions are essential. Common approaches include pathogen exposure, feed restriction, and chemical inducers. Among these, dextran sodium sulfate (DSS) is widely used for its ability to disrupt epithelial integrity, increase intestinal permeability, and activate mucosal immune responses, making it a relevant and reproducible tool for simulating enteric inflammation (Eichele and Kharbanda, 2017; Liu et al., 2022). In parallel, identifying reliable biomarkers is crucial for non-invasive monitoring of gut health. Calprotectin, a calcium-binding protein secreted by activated neutrophils, is well established as a marker of intestinal inflammation in mammals (Jukin et al., 2021). Its application in poultry is gaining traction, with recent studies showing elevated calprotectin levels in broilers challenged with DSS. These levels correlate with intestinal damage, supporting calprotectin's potential as a sensitive and practical indicator of gut barrier dysfunction and inflammation in poultry production systems (Dal Pont et al., 2021).

The aim of the present study is to investigate the effects of a synergistic blend of oregano, cinnamon, and clove essential oils on growth performance, intestinal morphology, carcass traits, and gut barrier function in broilers subjected to DSS-induced intestinal inflammation.

## Materials and methods

### Ethical endorsement

The experimental protocol (DK-1-2337), detailing the management and care of animals, was reviewed and approved by the Institutional Animal Care and Use Committee of Dankook University, South Korea.

### Birds, diets, and management

The experiment was conducted at the Experimental Facility of Dankook University, located in Sejong City, South Korea. The facility consisted of naturally ventilated, double-wing curtain housing equipped with three-tier battery cages (150 × 100 × 220 mm per cage). Each cage was fitted with a single-sided feeder and a nipple-type drinker, providing birds with ad libitum access to feed and water. Environmental conditions were controlled throughout the study, with a relative humidity of approximately 60 %, an 18-hour fluorescent light cycle, and a temperature program that began at 33°C and was gradually reduced by 3°C per week to reach 24°C by day 42.

A total of 840 one-day-old straight-run Ross 308 broiler chicks with an initial body weight of 44.47 ± 0.41 g were randomly allocated to two dietary treatments, each with 21 replicates and 20 birds per cage. The treatments consisted of: (1) a control group (CON) receiving a basal corn–soybean meal diet, and (2) a treatment group (FP) receiving the basal diet supplemented with 25 g/ton of Fytera Perform (Selko Feed Additives, Trouw Nutrition, The Netherlands). Fytera Perform is a microencapsulated phytogenic feed additive formulated with essential oils derived from oregano (*Origanum vulgare*), cinnamon (*Cinnamomum verum*), and clove (*Syzygium aromaticum*), providing a full spectrum of their naturally occurring phytoactive compounds.

Diets were formulated to meet or exceed the nutritional recommendations for Ross 308 broilers (Aviagen W, 2019) (Table 1) and were provided in three phases: starter (1–8 days), grower (9–21 days), and finisher (22–42 days).

**Table 1:** Ingredient and nutrient composition of the basal diets ( %, as-fed basis).

Ingredients, %	Starter	Grower	Finisher
	(0-8 days)	(9-21 days)	(22-42 days)
Corn	44.12	45.87	49.28
Wheat	10.00	10.00	10.00
Soybean meal	30.49	28.59	25.18
Distillers' dried grain soluble	5.00	5.00	5.00
Tallow	3.21	6.00	6.20
Soy oil	0.50	-	-
Limestone	1.42	1.06	1.08
Mono-dicalcium phosphate	1.88	1.66	1.58
Sodium bicarbonate	0.10	0.10	0.10
DL-Methionine, 99 %	0.51	0.42	0.43
Threonine 98.5 %	0.21	0.16	0.10
Choline,50 %	0.13	0.10	0.10
Salt	0.27	0.26	0.23
L-Lysine 50 %	2.00	0.62	0.56
Vitamin premix^1^	0.06	0.06	0.06
Mineral premix^2^	0.10	0.10	0.10
Calculated analysis			
Dry matter	87.31	87.53	87.57
Crude protein, %	22.00	20.50	18.50
Crude fat, %	5.74	7.85	8.05
Crude fiber, %	2.46	2.45	2.26
Crude ash, %	6.32	5.85	5.31
Metabolizable energy, kcal/kg	3030	3140	3250
Calcium, %	1.05	0.90	0.90
Available phosphorus	0.50	0.45	0.42
Lysine	2.04	1.34	1.12
Methionine	0.72	0.65	0.64
Cysteine	0.34	0.34	0.31
Threonine	0.97	0.91	0.79
Tryptophan	0.24	0.23	0.21
Methionine + cysteine	1.06	0.99	0.95
Digestible lysine	1.87	1.17	0.95
Digestible methionine	0.68	0.60	0.59
Digestible cysteine	0.26	0.26	0.25
Digestible threonine	0.83	0.76	0.64
Digestible tryptophan	0.20	0.20	0.19
Digestible methionine and cysteine	0.94	0.87	0.83

1 Provided per kg of complete diet: 11,025 IU vitamin A; 1103 IU vitamin D3; 44 IU vitamin E; 4.4 mg vitamin K; 8.3 mg riboflavin; 50 mg niacin; 4 mg thiamine; 29 mg d-pantothenic; 166 mg choline; 33 µg vitamin B12.

2 Provided per kg of complete diet: 12 mg Cu (as CuSO4:5H2O); 85 mg Zn (as ZnSO4); 8 mg Mn (as MnO2); 0.28 mg I (as KI); 0.15 mg Se (as Na2SeO35H2O).

### Intestinal challenge

To induce an enteric challenge, a 2.5 % DSS solution was administered via drinking water from day 22 to day 28. This period corresponds to the growth phase, during which the gastrointestinal tract is sufficiently developed to respond to dietary interventions and nutrient absorption is at its peak. The DSS concentration was selected based on previous studies demonstrating its ability to induce measurable intestinal disturbance without causing excessive mortality in broilers (Liu et al., 2022; Chen et al., 2023).

### Sampling and clinical analysis

#### Growth performance and mortality

Body weight (BW) and feed intake (FI) were recorded on days 0, 8, 21, and 42 to evaluate broiler performance. These data were used to calculate average daily gain (ADG), average daily feed intake (ADFI), and feed conversion ratio (FCR). Mortality was monitored daily.

#### Meat quality assessment

On day 28, a total of 84 birds (two per replicate) were randomly selected for the assessment of meat quality, intestinal morphology, and gene expression. After slaughter, breast muscles were excised within 15 minutes and immediately transported to the laboratory for analysis. The pH values of the breast muscle were measured using a digital pH meter (Fisher Scientific, Pittsburgh, PA, USA). Water-holding capacity (WHC) was assessed by applying 3 kg of pressure to 0.2 g of meat, followed by measurement of the area of water released. Drip loss was determined by weighing samples stored in airtight plastic bags at 4°C on days 1, 3, 5, and 7, and calculating the percentage of weight loss as described by Honikel (1987).

#### Carcass trait evaluation

On day 42, two birds per replicate were randomly selected and slaughtered for the analysis of carcass traits. After defeathering and evisceration, the breast and leg muscles, liver, heart, and gizzard were individually weighed and expressed as percentages of live body weight. The dressing percentage was calculated as the ratio of eviscerated carcass weight to live body weight.

#### Intestinal morphology

Intestinal morphology was evaluated by collecting the middle section of the ileum (between Meckel’s diverticulum and the ileocecal junction) from the same birds sampled on day 28. Samples were fixed in 4 % formaldehyde, embedded in paraffin, and sectioned at 5 μm using a rotary microtome (HM360, Thermo Scientific, Waltham, MA, USA). The sections were dewaxed with xylene, rehydrated through graded ethanol, stained with hematoxylin and eosin (H&E), and mounted on glass slides. For each sample, five well-oriented villus–crypt units were examined under a light microscope (Olympus Corporation, Tokyo, Japan) using ImageJ software (version 1.53). Villus height (VH) and crypt depth (CD) were measured, and the villus height-to-crypt depth ratio (VCR) was calculated.

#### Gene expression analysis

Ileal and cecal mucosa samples were collected for analysis of gene expressions related to inflammation and gut barrier integrity. Total RNA was extracted using TRIzol reagent (Invitrogen, Carlsbad, CA, USA), and complementary DNA (cDNA) was synthesized using the PrimeScript RT Reagent Kit (Takara Bio Inc., Shiga-Ken, Japan). Quantitative real-time PCR (qPCR) was performed using a CronoSTAR 96 Real-Time PCR System (Clontech, Mountain View, CA, USA). The target genes analyzed included interleukin-1 beta (IL-1β), interleukin-8 (IL-8), tight junction protein 1 (ZO-1), and mucin 2 (MUC2), with glyceraldehyde-3-phosphate dehydrogenase (GAPDH) serving as the reference gene. Relative gene expression levels were calculated using the 2^−ΔΔCt method (Table 2).

**Table 2:** Sequences of primers used for RT-PCR.

Gene^1^	Sequence	Size, bp	Annealing T,°C	Accession number
GAPDH	F: ACTGTCAAGGCTGAGAACGG	86	60	NM_204305.2
	R: CATTTGATGTTGCTGGGGTC			
IL-1β	F: CCAGCCAGAAAGTGAGGC	106	60	NM_204524.2
	R: TGTAGCCCTTGATGCCCA			
IL-8	F: TCTGTCGCAAGGTAGGACG	179	60	NM_205498.1
	R: AGCACACCTCTCTTCCATCC			
ZO1	F: GCCAACTGATGCTGAACCAA	141	60	XM_040706827.2
	R: GGGAGAGACAGGACAGGACT			
MUC2	F: TGAGTGTGAAGAATGCACCTG	169	65	XM_040701656.2
	R: ATCATTCACAGCACTCTTGGC			

1 GAPDH, Glyceraldehyde-3-phosphate dehydrogenase; ACTB, Actin, beta; IL-1b, Interleukin 1, beta; IL-8,Interleukin 8-like 2; ZO1, Tight junction protein 1; MUC2, mucin 2, oligomeric mucus/gel-forming.

#### Fecal calprotectin analysis

On day 28, fresh excreta samples were collected from two birds per replicate, immediately frozen in liquid nitrogen, and stored at −80°C. For analysis, 0.4 g of feces was mixed with 4 mL of phosphate-buffered saline, vortexed thoroughly, and centrifuged at 3,000 rpm for 20 minutes at 4°C. The resulting supernatant was then collected and stored at −20°C until further analysis. Calprotectin (avian MRP-126) concentrations were quantified using a commercial ELISA kit (MyBioSource Inc., San Diego, CA, USA) following the manufacturer’s instructions.

#### Data analysis

All experimental data were tested for normality and analyzed using the PROC MIXED procedure in SAS (SAS Institute, Cary, NC, USA). The model included treatment as a fixed effect and block as a random effect, with block defined as cage location. For growth performance parameters, including mortality rate, the cage was considered the experimental unit, whereas individual birds served as the experimental unit for meat quality, carcass traits, intestinal morphology, gene expression, fecal biomarkers, and pro-inflammatory markers. *P*-values less than or equal to 0.05 were considered significant, whereas *P-*values greater than 0.05 and less than or equal to 0.10 were considered a trend. Tukey Kramer’s level of significance was used to separate treatment means.

## Results

### Growth performance

Dietary supplementation with the synergistic blend of natural plant extracts (FP) significantly improved broiler growth performance during the finisher phase and over the entire production period (Table 3). During the starter-grower phase (0–21 days), no significant differences were observed between treatments for ADG, ADFI, or FCR (P > 0.05). However, during the finisher phase (22–42 days), birds in the FP group exhibited a significant increase in ADG (P = 0.015). FCR also improved in the FP group, with a trend towards significance (P = 0.052), while ADFI remained comparable between groups. Over the entire experimental period (0–42 days), FP supplementation resulted in a significantly higher ADG (62 g vs. 59 g; P = 0.001) and a 2.84 % improvement in FCR (1.468 vs. 1.511; P = 0.054). On day 42, final body weight was significantly greater in the FP group by 112 g (2,586 g vs. 2,474 g; P = 0.003). Additionally, mortality was reduced by 12.5 % in the FP group (3.92 %) compared to the CON group (4.48 %).

**Table 3:** The effect of phytogenic supplementation on growth performance of broilers.

Items	CON	FP	SEM	*P*-value
**Starter Phase (0-8 days)**				
ADG (g/d/bird)	24^y^	26^x^	0.400	0.085
ADFI (g/d/bird)	22.55	23.36	0.902	0.816
FCR, g/g	1.038	1.023	0.007	0.204
**Grower Phase (9-21 days)**				
ADG (g/d/bird)	56	58	1.100	0.173
ADFI (g/d/bird)	65.99	67.35	1.801	0.502
FCR, g/g	1.184	1.162	0.018	0.448
**Finisher Phase (22-42 days)**				
ADG (g/d/bird)	72^b^	75^a^	1.100	0.015
ADFI (g/d/bird)	124.10	125.76	2.261	0.236
FCR, g/g	1.733^y^	1.673^x^	0.026	0.052
**Overall Period (0-42 days)**				
Body weight, g	2474^b^	2586^a^	24.000	0.003
ADG (g/d/bird)	59^b^	62^a^	0.600	0.001
ADFI (g/d/bird)	87.31	88.73	1.158	0.803
FCR, g/g	1.511^y^	1.468^x^	0.017	0.054
Mortality, %	4.48	3.92	0.091	0.611

Within a row, means with different superscripts (a, b) differ significantly (P < 0.05); values with different superscripts (x–y) indicate a statistical trend (P < 0.10). Mean values are based on 20 birds per replicate and 21 replicates per treatment. CON, basal diet; FP, basal diet + Fytera Perform.

### Carcass traits and meat quality

FP supplementation positively affected specific carcass traits and meat quality parameters (Table 4, Table 5). As shown in Table 4, the dressing percentage tended to be higher in the FP group compared to the CON group (P = 0.07), while no significant differences were observed in the relative weights of breast, leg, gizzard, liver, or heart between treatments (P > 0.05).

**Table 4:** The effect of phytogenic supplementation on carcass traits of broilers on day 42.

Items	CON	FP	SEM	*P*-value
Dressing percentage, %	74.74^y^	76.13^x^	0.570	0.07
Breast, %	24.69	25.83	0.658	0.22
Leg, %	21.28	21.42	0.383	0.80
Gizzard, %	2.32	2.22	0.039	0.11
Liver, %	5.40	5.44	0.093	0.77
Heart, %	1.06	1.05	0.016	0.61

Within a row, means with different superscripts (x-y) indicate a statistical trend (P < 0.10). Mean values are based on 2 birds per replicate and 21 replicates per treatment. CON, basal diet; FP, basal diet + Fytera Perform.

**Table 5:** The effect of phytogenic supplementation on the meat quality of broilers on day 28.

Items	CON	FP	SEM	*P*-value
pH	5.35	5.55	0.080	0.12
Water holding capacity, %	48.75	51.02	0.950	0.15
Drip loss, %				
d1	1.52	1.44	0.060	0.37
d3	4.08	3.94	0.060	0.11
d5	6.09^a^	5.91^b^	0.060	0.03
d7	8.21^a^	7.95^b^	0.030	0.01

Within a row, means with different superscripts (a-b) differ significantly (P < 0.05). Mean values are based on two birds per replicate and 21 replicates per treatment. CON, basal diet; FP, basal diet + Fytera Perform.

Regarding meat quality (Table 5), drip loss did not differ significantly between treatments on days 1 (P = 0.37) and 3 (P = 0.11). However, drip loss was significantly reduced in the FP group on days 5 (5.91 % vs. 6.09 %; P = 0.03) and 7 (7.95 % vs. 8.21 %; P = 0.01). No treatment effects were observed for pH or WHC (P > 0.05).

### Intestinal morphology

Data on intestinal histomorphology showed significant improvements with FP supplementation (Table 6). VH was significantly greater in the FP group compared to CON (P = 0.002). CD tended to be lower in the FP group (P = 0.083), contributing to a significant increase in the VCR in the FP group (P = 0.018).

**Table 6:** The effect of phytogenic supplementation on ileum morphology at day 28.

Item	CON	FP	SEM	P-value
Villus height, µm	593.60^b^	641.74^a^	10.210	0.002
Crypt depth, µm	207.71^x^	195.33^y^	4.920	0.083
Villi height: Crypt ratio	2.90^b^	3.40^a^	0.130	0.018

Within a row, values with different superscripts (a–b) differ significantly (P < 0.05); values with different superscripts (x–y) indicate a statistical trend (P < 0.10). Mean values are based on 2 birds per replicate and 21 replicates per treatment. CON, basal diet; FP, basal diet + Fytera Perform

### Gene expression of barrier function markers

**Fig 1:**
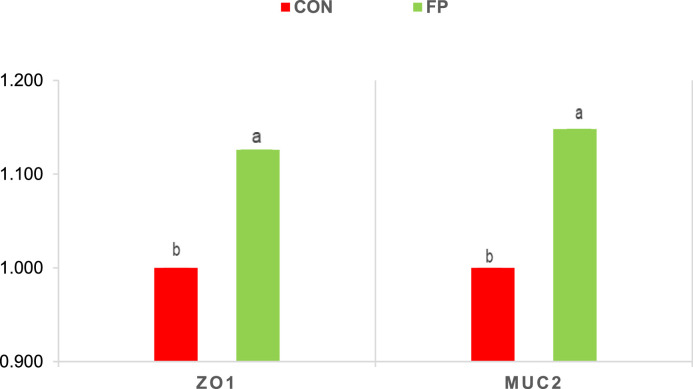
Effect of phytogenic supplementation on ZO1 and MUC2 gene expression in the cecal mucosa. CON, basal diet; FP, basal diet + Fytera Perform. Bars without a common superscript differ significantly (P<0.05).

**Fig 2:**
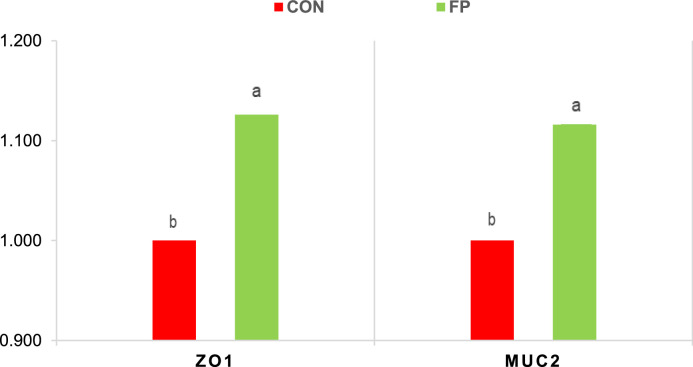
Effect of phytogenic supplementation on ZO1 and MUC2 gene expressions in the ileum mucosa. CON, basal diet; FP, basal diet + Fytera Perform. Bars without a common superscript differ significantly (P<0.05).

Dietary supplementation with FP significantly enhanced the expression of genes associated with mucosal barrier integrity. In the ileum, the expression levels of MUC2 and ZO-1 were significantly higher in the FP group compared to the CON group (P = 0.01 for both) (Fig. 1). A similar pattern was observed in the cecum, where FP supplementation led to significant upregulation of both MUC2 and ZO-1 gene expression (P = 0.002 and P = 0.006, respectively)) (Fig. 2).

### Inflammatory markers

**Fig 3:**
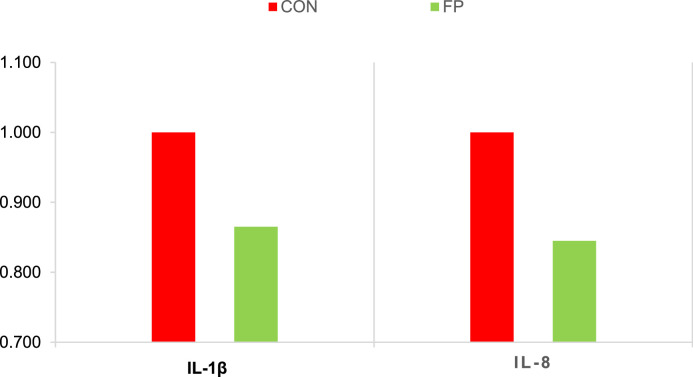
Effect of phytogenic supplementation on IL-1β and IL-8 gene expression in the ileum tissue. CON, basal diet; FP, basal diet + Fytera Perform. Bars without a common superscript differ significantly (P<0.05).

**Fig 4:**
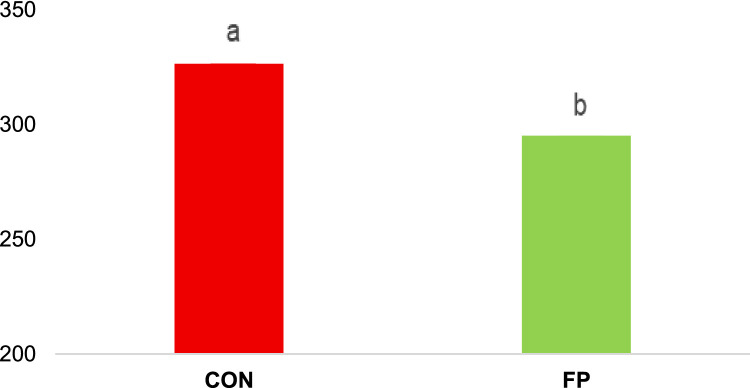
Effect of phytogenic supplementation on fecal biomarkers in broilers. CON, basal diet; FP, basal diet + Fytera Perform. Bars without a common superscript differ significantly (P<0.05).

Supplementation with FP significantly attenuated inflammatory responses in broiler chickens. Expression levels of the pro-inflammatory cytokines IL-1β and IL-8 were markedly lower in the FP group compared to CON (P = 0.01 and P = 0.0003, respectively; Fig. 3). In addition, the concentration of calprotectin was significantly reduced in the FP-supplemented group (P = 0.046; Fig. 4).

## Discussion

The results of this trial demonstrate that supplementation with the phytogenic blend (FP) significantly improved ADG and FCR throughout the production period, with the most pronounced effects observed during the finisher phase (days 22 to 42). These delayed yet significant improvements, particularly under DSS-induced stress, suggest that FP initiated physiological changes early in development that enhanced performance later. While growth differences during the starter and grower phases were modest, early supplementation may have modulated intestinal barrier integrity, immune function, and microbial stability (Kogut, 2019; Al-Garadi et al., 2025; Ceylan et al., 2025). These adaptations likely contributed to improved resilience, as reflected in better growth, feed efficiency, and reduced mortality in the FP group during the finisher phase (Zhang et al., 2025). These findings align with the progressive maturation of the gastrointestinal tract and microbial ecosystem in broilers. In the early post-hatch period, the gut is still developing, and the microbial community is not fully established, which may limit the immediate impact of functional feed additives (Yegani and Korver, 2008; Apajalahti and Vienola, 2016). Nonetheless, early exposure to FP may prime gut development, leading to enhanced digestive enzyme activity, improved barrier function, and more efficient nutrient absorption as the birds mature (Choct, 2009; Gao et al., 2017). Although feed intake was not significantly different, the improved FCR in birds receiving FP suggests better digestion and nutrient use in the later stages of production.

Histomorphology analyses revealed that FP supplementation significantly increased VH and the VCR, indicating improved mucosal architecture and nutrient absorption under enteric stress. These structural changes are consistent with enhanced epithelial cell turnover and regeneration, which are critical for maintaining effective nutrient uptake and barrier function during periods of intestinal challenge (Ducatelle et al., 2018). These findings corroborate previous reports demonstrating that phytogenic feed additives containing oregano, thyme, or cinnamon essential oils exert protective effects on gut morphology by promoting epithelial cell proliferation, reducing oxidative damage, and modulating inflammatory responses (Gopalsamy et al., 2025; Puvaca et al., 2022).

At the molecular level, FP supplementation enhanced gut barrier integrity, evidenced by upregulation of the tight junction protein ZO-1 and the mucin gene MUC2 in both the ileum and cecum. Tight junction proteins, including ZO-1, play a fundamental role in sealing intercellular spaces and preventing paracellular leakage, while mucins such as MUC2 form a protective mucus layer that limits microbial adherence and translocation (Ismail et al., 2023; Pan et al., 2023;). The observed upregulation of these genes suggests that FP promotes maintenance and restoration of epithelial integrity under inflammatory stress. This was accompanied by reduced expression of pro-inflammatory cytokines IL-1β and IL-8, which are typically elevated during DSS-induced intestinal injury (Oni et al., 2023; Wang et al., 2024). Moreover, fecal calprotectin, a non-invasive biomarker of intestinal inflammation, was significantly decreased in the FP group, further indicating attenuated mucosal inflammation and barrier disruption. Together, these molecular and histological data provide strong evidence that FP supplementation supports mucosal homeostasis and mitigates inflammatory damage.

The beneficial effects of FP on intestinal health may be mediated through modulation of intracellular signaling pathways involved in immune and oxidative responses. Bioactive compounds present in FP, such as carvacrol, thymol, cinnamaldehyde, and eugenol, have been shown to regulate the NF-κB and MAPK pathways, leading to reduced pro-inflammatory cytokine production and oxidative stress (Windisch et al., 2008; Murugesan et al., 2015). Previous studies have also demonstrated that essential oils from oregano and clove improve tight junction integrity and reduce intestinal permeability, consistent with the present observations (Zhang et al., 2023). However, the exact molecular targets and dose-response relationships warrant further investigation.

Beyond intestinal effects, FP supplementation positively influenced meat quality traits. The reduction in drip loss on storage days 5 and 7 indicates improved water-holding capacity, an important factor for meat juiciness, appearance, and consumer acceptability (Sampath et al. 2023). This improvement may be attributed to bioactive compounds in FP, which stabilize cell membranes and preserve muscle structure, thereby reducing water loss (Cicalau et al., 2021; Pei et al., 2023). These results correspond with previous reports showing that phytogenic additives enhance meat quality by modulating muscle cell integrity and antioxidant pathways, thus reducing exudative losses (Pei et al., 2023). Additionally, the FP group showed a trend toward increased dressing percentage, potentially reflecting improved metabolic efficiency; however, further research is needed to confirm this effect.

## Conclusion

This study demonstrates that dietary supplementation with a synergistic blend of oregano, clove, and cinnamon extracts (FP) effectively mitigates intestinal inflammation in broilers. FP modulated inflammatory responses, improved intestinal morphology, and strengthened gut barrier integrity, resulting in enhanced nutrient absorption, growth performance and meat quality. These findings support FP supplementation as a promising nutritional strategy to improve gut health and productivity in broilers under intestinal stress.

## CRediT authorship contribution statement

**Vetriselvi Sampath:** Writing – original draft, Software, Investigation, Formal analysis, Data curation. **Lane Pineda:** Writing – review & editing, Writing – original draft, Investigation, Conceptualization. **Ellen Hambrecht:** Writing – review & editing, Validation, Methodology, Conceptualization. **In Ho Kim:** Validation, Supervision, Project administration, Methodology, Conceptualization.

## Disclosures

Regarding the information presented in the manuscript, we confirm that there are no financial organizations with which we have a conflict of interest.
